# Supplementary material for: Unveiling the Bioactive Potential of the Invasive Jellyfish Phyllorhiza punctata Through Integrative Transcriptomic and Proteomic Analyses
Source: Biomolecules. 2025 Aug 4;15(8):1121. doi: 10.3390/biom15081121 (PMC12383608; doi:10.3390/biom15081121)
Supplement: Supplementary file 1 [file biomolecules-15-01121-s001.zip › Suplementary Material/FileS3/PEPFOLD4/XP_065054022_7248/clusters.html]

 PEP-FOLD clusters


Download archive of all models  here   

|  |  |  |
| --- | --- | --- |
| # | file | sOPEP |
| # cluster 1 - Size: 70 |  |  |
|  | XP\_065054022\_7248-model1 | -9.86223 |
|  | XP\_065054022\_7248-model1.1 | -9.86223 |
|  | XP\_065054022\_7248-model1.2 | -9.86223 |
|  | XP\_065054022\_7248-model1.3 | -9.86223 |
|  | XP\_065054022\_7248-model1.4 | -9.86223 |
|  | XP\_065054022\_7248-model1.5 | -9.86223 |
|  | XP\_065054022\_7248-model1.6 | -9.86223 |
|  | XP\_065054022\_7248-model1.7 | -9.86223 |
|  | XP\_065054022\_7248-model1.8 | -9.86223 |
|  | XP\_065054022\_7248-model1.9 | -9.86223 |
|  | XP\_065054022\_7248-model1.10 | -9.86223 |
|  | XP\_065054022\_7248-model1.11 | -9.86223 |
|  | XP\_065054022\_7248-model1.12 | -9.86223 |
|  | XP\_065054022\_7248-model1.13 | -9.86223 |
|  | XP\_065054022\_7248-model1.14 | -9.86223 |
|  | XP\_065054022\_7248-model1.15 | -9.86223 |
|  | XP\_065054022\_7248-model1.16 | -9.86223 |
|  | XP\_065054022\_7248-model1.17 | -9.86223 |
|  | XP\_065054022\_7248-model1.18 | -9.86223 |
|  | XP\_065054022\_7248-model1.19 | -9.86223 |
|  | XP\_065054022\_7248-model1.20 | -9.86223 |
|  | XP\_065054022\_7248-model1.21 | -9.86223 |
|  | XP\_065054022\_7248-model1.22 | -9.86223 |
|  | XP\_065054022\_7248-model1.23 | -9.86223 |
|  | XP\_065054022\_7248-model1.24 | -9.86223 |
|  | XP\_065054022\_7248-model1.25 | -9.86223 |
|  | XP\_065054022\_7248-model1.26 | -9.86223 |
|  | XP\_065054022\_7248-model1.27 | -9.86223 |
|  | XP\_065054022\_7248-model1.28 | -9.86223 |
|  | XP\_065054022\_7248-model1.29 | -9.86223 |
|  | XP\_065054022\_7248-model1.30 | -9.67852 |
|  | XP\_065054022\_7248-model1.31 | -9.67852 |
|  | XP\_065054022\_7248-model1.32 | -9.67852 |
|  | XP\_065054022\_7248-model1.33 | -9.67852 |
|  | XP\_065054022\_7248-model1.34 | -9.67852 |
|  | XP\_065054022\_7248-model1.35 | -9.67852 |
|  | XP\_065054022\_7248-model1.36 | -9.67852 |
|  | XP\_065054022\_7248-model1.37 | -9.67852 |
|  | XP\_065054022\_7248-model1.38 | -9.67852 |
|  | XP\_065054022\_7248-model1.39 | -9.67852 |
|  | XP\_065054022\_7248-model1.40 | -9.67852 |
|  | XP\_065054022\_7248-model1.41 | -9.67852 |
|  | XP\_065054022\_7248-model1.42 | -9.67852 |
|  | XP\_065054022\_7248-model1.43 | -9.67852 |
|  | XP\_065054022\_7248-model1.44 | -9.67852 |
|  | XP\_065054022\_7248-model1.45 | -9.67852 |
|  | XP\_065054022\_7248-model1.46 | -9.67852 |
|  | XP\_065054022\_7248-model1.47 | -9.67852 |
|  | XP\_065054022\_7248-model1.48 | -9.67852 |
|  | XP\_065054022\_7248-model1.49 | -9.67852 |
|  | XP\_065054022\_7248-model1.50 | -9.67852 |
|  | XP\_065054022\_7248-model1.51 | -9.67852 |
|  | XP\_065054022\_7248-model1.52 | -9.67852 |
|  | XP\_065054022\_7248-model1.53 | -9.67852 |
|  | XP\_065054022\_7248-model1.54 | -9.67852 |
|  | XP\_065054022\_7248-model1.55 | -9.67852 |
|  | XP\_065054022\_7248-model1.56 | -9.67852 |
|  | XP\_065054022\_7248-model1.57 | -9.67852 |
|  | XP\_065054022\_7248-model1.58 | -9.61573 |
|  | XP\_065054022\_7248-model1.59 | -9.61573 |
|  | XP\_065054022\_7248-model1.60 | -9.61573 |
|  | XP\_065054022\_7248-model1.61 | -9.61573 |
|  | XP\_065054022\_7248-model1.62 | -9.61573 |
|  | XP\_065054022\_7248-model1.63 | -9.61573 |
|  | XP\_065054022\_7248-model1.64 | -9.61573 |
|  | XP\_065054022\_7248-model1.65 | -9.61573 |
|  | XP\_065054022\_7248-model1.66 | -9.61573 |
|  | XP\_065054022\_7248-model1.67 | -9.61573 |
|  | XP\_065054022\_7248-model1.68 | -9.5957 |
|  | XP\_065054022\_7248-model1.69 | -9.5594 |
| # cluster 2 - Size: 28 |  |  |
|  | XP\_065054022\_7248-model2 | -4.94797 |
|  | XP\_065054022\_7248-model2.1 | -4.94797 |
|  | XP\_065054022\_7248-model2.2 | -4.94797 |
|  | XP\_065054022\_7248-model2.3 | -4.83661 |
|  | XP\_065054022\_7248-model2.4 | -4.78089 |
|  | XP\_065054022\_7248-model2.5 | -4.78089 |
|  | XP\_065054022\_7248-model2.6 | -4.74547 |
|  | XP\_065054022\_7248-model2.7 | -4.73399 |
|  | XP\_065054022\_7248-model2.8 | -4.65914 |
|  | XP\_065054022\_7248-model2.9 | -4.65914 |
|  | XP\_065054022\_7248-model2.10 | -4.48369 |
|  | XP\_065054022\_7248-model2.11 | -4.47973 |
|  | XP\_065054022\_7248-model2.12 | -4.45989 |
|  | XP\_065054022\_7248-model2.13 | -4.43951 |
|  | XP\_065054022\_7248-model2.14 | -4.37337 |
|  | XP\_065054022\_7248-model2.15 | -4.34902 |
|  | XP\_065054022\_7248-model2.16 | -4.34902 |
|  | XP\_065054022\_7248-model2.17 | -4.34024 |
|  | XP\_065054022\_7248-model2.18 | -4.29539 |
|  | XP\_065054022\_7248-model2.19 | -4.29539 |
|  | XP\_065054022\_7248-model2.20 | -4.29539 |
|  | XP\_065054022\_7248-model2.21 | -4.29539 |
|  | XP\_065054022\_7248-model2.22 | -4.29539 |
|  | XP\_065054022\_7248-model2.23 | -4.29539 |
|  | XP\_065054022\_7248-model2.24 | -4.27233 |
|  | XP\_065054022\_7248-model2.25 | -4.0225 |
|  | XP\_065054022\_7248-model2.26 | -3.961 |
|  | XP\_065054022\_7248-model2.27 | -3.27178 |
| # cluster 3 - Size: 1 |  |  |
|  | XP\_065054022\_7248-model3 | -4.21979 |
| # cluster 4 - Size: 1 |  |  |
|  | XP\_065054022\_7248-model4 | -2.49319 |
|  |  |  |
